# HIV epidemic amidst COVID-19 pandemic in India: a conundrum for the country's healthcare system

**DOI:** 10.1017/S095026882200098X

**Published:** 2022-05-26

**Authors:** Ishita Ray, Mohammad Mehedi Hasan, Pritik A Shah, Abdul Moiz Sahito, Anusua Sarkar, Diya Ghosh, Mainak Bardhan, Nobendu Mukerjee, Mohammad Yasir Essar

**Affiliations:** 1 Mahatma Gandhi Memorial Medical College, Indore, India; 2 Department of Biochemistry and Molecular Biology, Faculty of Life Science, Mawlana Bhashani Science and Technology University, Tangail, Bangladesh; 3 Bangalore Medical College and Research Institute, Bangalore, India; 4 Dow University of Health Sciences, Karachi, Pakistan; 5 Department of Biotechnology, Heritage Institute of Technology, Kolkata, India; 6 Division of Bacteriology, ICMR-National Institute of Cholera and Enteric Diseases, Kolkata, India; 7 Department of Microbiology, Ramakrishna Mission Vivekananda Centenary College, Kolkata, India; 8 Kabul University of Medical Sciences, Kabul, Afghanistan

**Keywords:** COVID-19, healthcare system, HIV, India, public health

## Abstract

India has the third-largest burden of human immunodeficiency virus (HIV) infection in the world. The coronavirus disease 2019 (COVID-19) pandemic has only exposed the cracks in the Indian healthcare infrastructure concerning HIV. The prevalence of HIV in India is more among the destitute or sections of society shrouded by years of social stigma such as prostitutes, truck drivers, transsexuals and intravenous drug users. National AIDS Control Organisation and The Joint United Nations Programme on HIV/AIDS (UNAIDS) organisation have many several efforts over the years to set up counselling and testing centres all over the country and spread awareness about HIV among the masses. COVID-19 pandemic has reversed years of progress made by the same. HIV patients are biologically more susceptible to COVID-19, and the lockdown has led to the loss of jobs, economic crises, shortage of drugs and necessities such as food and housing among this vulnerable population, which can result in lowered CD4-T cell counts in the coming months and make way for dangerous opportunistic infection outbreaks in this population increasing the overall HIV burden of India. This article explores how COVID-19 has impacted India's already existing HIV epidemic and tries to put forth recommendations based on the evidence found to be better prepared in treating the HIV-positive population in India in the face of another catastrophe like the COVID-19.

## Introduction

Human immunodeficiency virus (HIV), the third widespread epidemic in the world, is a huge burden to the lower-middle-income country (LMIC), India. HIV prevalence among adults between the ages of 15 and 49 years was estimated to be 0.2% in 2017. Although the number is lesser than other LMICs, India's population, estimated at 1.3 billion, manifests to 2.1 million people living with the disease [1]. Several issues, including the stigma associated with HIV, relatively low awareness of the status of people with HIV and a broken link between diagnosis and treatment, indicate that progress is slower than expected. The lack of information on key populations and certain key indicators, such as virus suppression rates, also makes it tedious to design health programmes for HIV patients to cater to the needs of those amongst the HIV epidemic of the country [2].

As an additive to these concerns is the coronavirus disease 2019 (COVID-19) pandemic, which serves as a dependent factor breaking the already-existing gap between the HIV epidemic and the fragile Indian healthcare system. The Director of Post Graduate Institute of Medical Education and Research, Chandigarh, on 13 September 2021, stated that India is at the beginning of COVID-19 next wave, which was predicted to target the younger age groups. However, a serosurvey study conducted with a population of 2700 children showed that 71% had developed antibodies. This probably indicates that youngsters might not be affected disproportionately during the next wave [3].

The increase in HIV cases is not irrational since numerous infectious diseases, including tuberculosis, fungal mucormycosis and Zika, have been on the rise in the current pandemic in India. HIV might exhibit a similar tendency, worrying that the healthcare system is already weakened [4–6]. COVID-19 has a distinct impact on people living with HIV (PLHIV), and its effect on HIV research and therapy will continue long after the COVID-19 crisis has passed [7]. Many experts initially thought that PLHIV were particularly vulnerable to SARS-CoV-2 infection because they had a higher burden of some comorbidities, higher systemic inflammation despite effective antiretroviral therapy (ART) and some degree of immune alteration even among those on effective ART with immune reconstitution [8, 9]. A San Francisco research of 4252 PLHIV found that PLHIV were more vulnerable to SARS-CoV-2 infection than individuals who did not have HIV [10]. Inequality in access to healthcare and education disproportionately impacts impoverished communities, and COVID-19 negatively impacts those living with HIV. With this succinct message, we propose a mechanism for contextualizing the impact of COVID-19 on PLHIV. The purpose of this article is to (a) describe the associations between the COVID-19 and HIV epidemics; (b) describe the role it will play in the future with its effects on PLHIV; and (c) formulate an action plan for researchers and medical communities to react to the effects of the above mentioned, including prevention and treatment plans.

## Pre pandemic efforts taken against HIV by India

National AIDS Control Organisation (NACO) is the body that manages HIV control in India. NACO aims to extend medical therapy to all PLHIV in India. They have been making consistent efforts to abolish the stigma around HIV patients and create awareness about the fundamental modes of spreading HIV. They envision building Integrated Counselling & Testing Centres (ICTCs) over the country (including remote areas). Hence, every HIV patient has access to quality care and an opportunity to live a safe and healthy life. India provides antiretroviral drugs free of cost [11]. Free distribution of condoms among vulnerable groups is another method to reduce HIV spread [11]. These centres are also essential in transparently calculating the prevalence of HIV in India. Pregnant women with HIV have been a significant target of ICTC to help bring HIV-free babies to the world. NACO forms partnerships with many non-governmental organisations, faith-based organisations and women's self-help organisations to make their vision a reality [11].

## Challenges of COVID-19 to India's efforts against HIV

According to official figures, India has a staggering over 2.3 million PLHIV/acquired immune deficiency syndrome (AIDS), making it the world's third most heavily affected country by this dangerous sickness. The disease is centred mostly among disadvantaged communities, such as sex workers, who are disproportionately affected in India. In addition to men having sexual relations, those who use intravenous drugs are at risk [1], as are migratory labourers, truck drivers and transgender people (hijras). According to one study [1], a lack of sex education and safe sex behaviours can potentially raise the risk of HIV transmission.

India still fails to establish policies to manage its HIV population, and COVID-19 has only added fuel to the fire. COVID-19 has created a significant burden on this vulnerable HIV-positive population struggling with financial aid, obtaining medical care, and seeking help for their psychological problems like anxiety, depression and others. HIV-positive people are all more prone, just like the average population [12]. The vulnerable populations like sex workers have no source of income during the lockdown, and they already have been subjected to stigma and are reluctant to seek medical therapy for HIV. Migrant workers and truck drivers are also facing unemployment due to the COVID-19 pandemic. Loss of jobs and layoffs significantly increased during the pandemic, and many HIV-positive patients faced severe economic crises [1].

AIDS-affected states Maharashtra and Andhra Pradesh are the most severe in India, and they were also the hardest hit by the COVID-19 pandemic, which occurred in the same year. By July 2020, there would have been 570 antiretroviral treatment centres and 1264 link antiretroviral treatment centres operating under NACO; however, due to COVID-19's state-wide lockdown, the vulnerable population would not have sufficient access to these services [13].

HIV/AIDS patients are more susceptible to COVID-19 infection, plus the lockdown creates trouble in accessing food, medicines, etc. All the resources and infrastructure initially devoted to managing HIV/AIDS have been deployed for battling COVID-19 [12]. HIV/AIDS patients need access to treatment to ensure that their viral load remains undetectable and their CD4 count is high enough to protect them from HIV and COVID-19 [14]. HIV-positive people face many stigmas in society regardless of the pandemic, and many of them prefer to hide their HIV status; hence it is difficult for them to seek help and support during the tough times of the pandemic [15].

## UNAIDS and their recommendations during COVID-19

The Joint United Nations Programme on HIV/AIDS (UNAIDS) is leading the global effort to eliminate HIV/AIDS as a public health issue by 2030 as part of the Sustainable Development Goals [15]. By 2020, the UNAIDS goal is to achieve 90:90:90. According to this, 90% of PLHIV should be aware of their HIV status, 90% of positive patients should begin antiretroviral medication (ART) immediately, and 90% of those on ART should be virally suppressed by 2020 [11]. COVID-19 infections are more likely to infect older PLHIV patients and those with heart or lung disease. During lockdowns, UNAIDS recommends that persons living with HIV contact local authorities as soon as possible to ensure that they receive multi-month renewals of ART prescriptions. Both PLHIV and individuals from underserved groups at risk of contracting HIV should continue to use condoms, sterile needles and syringes for at-risk populations, pre-exposure prophylaxis and HIV testing [16].

Despite the efforts of the NACO, only 76% of the PLHIV know their status, and only 56% of them are under ART. The HIV epidemic is mainly concentrated among the key population groups. HIV self-testing is an excellent approach where people with HIV collect their samples (blood or saliva), perform a test and interpret the results. HIV self-test can undoubtedly be helpful during the COVID-19 pandemic; however, awareness about it in India is still low [11].

## Future recommendations

This is a watershed moment in the history of HIV/AIDS. This lethal disease is attracting unprecedented money, political will and media attention. The World Health Report 2004 states that the global community has a once-in-a-lifetime opportunity to change the course of history by augmenting existing prevention measures and upgrading health systems with HIV treatment programmes [17]. According to data, HIV treatment programmes were successful in 2020, with the number of people taking antiretroviral medication growing by 9% from 20.1 million in 2019 to 21.9 million in 2020. However, critical prevention and testing programmes fell short of expectations for the first time in the Global Fund's existence [18]. As the COVID-19 epidemic spreads, it is uncertain whether primary treatment and preventative programmes delays will cause countries like India to fall further behind. To avoid long-term devastation in the HIV/AIDS fight, we must rapidly increase adaptation and mitigation efforts to reclaim lost territory. Collecting reliable data on the volume, trends and distribution of HIV and AIDS illness is critical to effective control and prevention of the AIDS pandemic. These serve as the foundation for developing and implementing successful interventions. This is a crucial consideration that must constantly be taken into account while planning future policies and implementations. Because India is a heavily populated country, separating PLHIV into tiny clusters and pockets makes relief operations easier. In these clusters, rigorous and frequent testing should be performed, and if somebody is found as having HIV infection, their contacts should be tested to confirm the presence of the virus [19]. Safe and sanitised methods, particularly in the under-researched category of barbers and street-based tooth extractors, should be prioritised by requiring them to discard the blades after use. Strict standards requiring the use of safe and disposable syringes should be implemented. Weekly audits should be performed to ensure that these procedures are followed. Otherwise, only counselling will be ineffective in this demographic segment [19]. Early identification of the problem is required, followed by the development of comprehensive strategic frameworks for intervention. Mechanisms for coordinating a multi-sectorial collaborative plan must be an inherent element of the country's national HIV/AIDS strategy.

## Conclusion

India's population can make a difference today and in the future by working together and creating awareness about the critical but still underserved issue of HIV-AIDS. HIV requires society to tackle personal, often taboo, subjects openly and honestly. Thanks to courageous and determined activists, community organisers, health experts and their collaborations with NACO, the barriers are gradually crumbling. As the fight against HIV/AIDS proceeds, India will face a slew of both expected and unforeseen problems. Perseverance and resilience and efficacy, and grit are required at this vital juncture in history. The elimination of this crippling disease is not an insurmountable feat if the entire nation works together.

## Data Availability

Data sharing is not applicable to this article as no new data were created or analysed in this study.
